# Rare presentation of Rothmund-Thomson syndrome with novel compound heterozygous mutations of the RECQL4 gene^☆^^☆☆^

**DOI:** 10.1016/j.abd.2019.10.006

**Published:** 2020-05-14

**Authors:** Xinyue Zhang, Songmei Geng, Yi Zheng

**Affiliations:** Department of Dermatology, Second Affiliated Hospital, Xi’An Jiaotong University, Shaanxi, China

Dear Editor,

Rothmund-Thomson syndrome (RTS) is a rare autosomal recessive disorder that is characterized by facial rash (poikiloderma, a diagnostic hallmark), growth retardation, sparse scalp hair/eyelashes/eyebrows, juvenile cataracts, skeletal abnormalities, radial ray defects, and a predisposition to cancer. There are two clinical forms: type I, which is characterized by poikiloderma, ectodermal dysplasia, and juvenile cataracts with unknown etiology, and type II which is characterized by poikiloderma, congenital bone defects, an increased frequency of malignancy (especially osteosarcoma), and RECQL4 (8q24.3) mutation.^1^ To date, around 400 cases have been reported.

Here, the authors report a case of poikiloderma and growth retardation in a Chinese girl presenting two RECQL4 mutations in a novel, compound heterozygous arrangement (c.2492_2493del and c.1391-2A>C) recorded *via* mutational screening, which is the first reported in RTS.

The proband is a 2-year-old girl with poikiloderma bilaterally on her face and ears. Her parents complained that their younger daughter showed erythema, swelling, and blistering bilaterally on her face since the age of 6 months, which gradually developed to reticulated hypo- and hyperpigmentation. The girl also presented with thinning of eyebrows, photosensitivity, and gastrointestinal problems including chronic emesis or diarrhea. Neither her parents nor her 5-year-old sister has similar symptoms. The patient was born at full term with a mild toe abnormality. However, slow weight gain, short stature, and teeth retardation were noted on a physical examination. The dermatological examination found bilateral depigmentation, hyperpigmentation, punctate atrophy, and telangiectasia over the patient's face and ears (Fig. 1). Bone mineral density measurement was performed at 1 year of age, which showed low bone mineral density. Her cognitive ability, ophthalmic testing, and other examination results were within normal limits and no other alterations were found.

**Figure 1:**
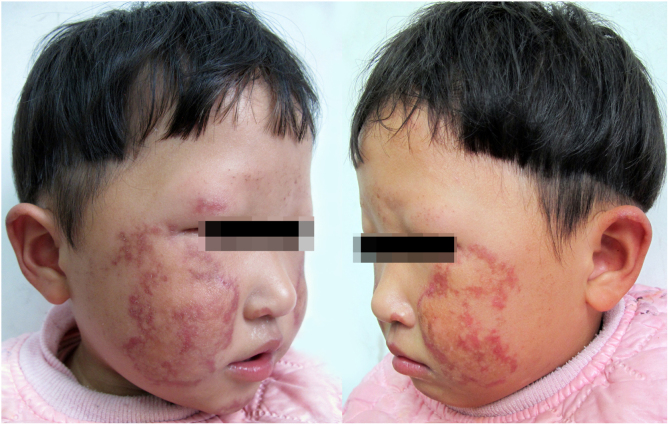
Poikiloderma in a patient with RTS. Depigmentation, hyperpigmentation, punctate atrophy, telangiectasia, and loss of eyebrows are seen bilaterally on the face.

For differential molecular diagnosis of poikiloderma, targeted exome sequencing was performed. Mutational screening for BLM, the defective gene in Bloom's syndrome and other poikiloderma-related diseases, was negative. Gene sequencing revealed two distinct heterozygous mutations on the RECQL4 gene (Fig. 2). One of them is a point mutation located in exon 9, consisting of a change of adenine for cytosine (c.1391-2A>C), which was found in her unaffected father and sister. This mutation has not been reported, but the possible effect on the protein through a splice acceptor variant can be assumed. On the other allele, the mutation is a deletion of two nucleotides found in exon 16 (c.2492_2493delAT), which produces a frame shift (p.His831Argfs); this mutation is known to be rare and last evaluated by Kitao et al.^2^ This mutation was found in her unaffected mother. These two mutations of the proband respectively come from her father and mother, known as compound heterozygous mutations, and accord with the autosomal recessive inheritance law. Her sister only presents c.1391-2A>C, which is a heterozygous mutation and, theoretically, she won’t show any symptoms.

**Figure 2:**
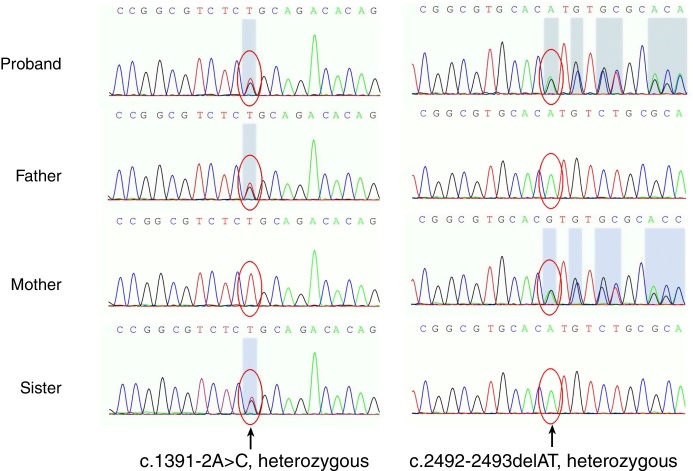
Two novel heterozygous variants in the RECQL4 gene confirmed by gene sequencing. One was in the splice site, c.1391-2A>C from her father, and also was seen in her sister. The other was a deletion mutation, c.2492_2493delAT (p.His831Argfs) from her mother.

The patient reported here has the clinical signs like poikiloderma, sparse eyebrows, small stature, dental abnormality, and mild skeletal abnormality, which are mentioned in the previous articles.^3^, ^4^ No cataracts and cancer have been found so far. Unlike other previous cases, the lesion doesn’t affect her extremities, and the authors consider that she is too young to show all the symptoms. RTS was diagnosed according to typical lesion and mutation of RECQL4 gene, and the patient was advised to avoid sun exposure and undergo annual checkups for the eyes, skin, and bones.

The novels compound heterozygous RECQL4 mutations presented in this patient is the first reported in RTS. Loss of RECQL4 protein function occurs in approximately two-thirds of RTS patients and is associated with risk of osteosarcoma.^5^ Further functional studies to confirm the protein-damaging effect are needed to proceed. Poikiloderma is a symptom of many systemic diseases, such as lupus erythematosus, Bloom syndrome, Kindler syndrome, and dyskeratosis congenita. The result of genetic testing is instructive and meaningful to a definitive diagnosis and future procreation guidance for the patient's family.

## Financial support

None declared.

## Authors’ contributions

Xinyue Zhang: Drafting and editing of the manuscript.

Songmei Geng: Approval of final version of the manuscript; intellectual participation in the propaedeutic and/or therapeutic conduct of the studied cases.

Yi Zheng: Critical review of the literature.

## Conflicts of interest

None declared.

## References

[1] Larizza L., Roversi G., Volpi L. (2010). Rothmund-Thomson syndrome. Orphanet J Rare Dis.

[2] van Rij M.C., Grijsen M.L., Appelman-Dijkstra N.M., Hansson K.B., Ruivenkamp C.A., Mulder K. (2017). Rothmund-Thomson syndrome and osteoma cutis in a patient previously diagnosed as COPS syndrome. Eur J Pediatr.

[3] Larizza L., Roversi G., Verloes A. (2013). Clinical utility gene card for: Rothmund-Thomson syndrome. Eur J Hum Genet.

[4] Yang J.Y., Sohn Y.B., Lee J.S., Jang J.H., Lee E.S. (2017). Rare presentation of Rothmund-Thomson syndrome with predominantly cutaneous findings. JAAD Case Rep.

[5] Siitonen H.A., Sotkasiira J., Biervliet M., Benmansour A., Capri Y., Cormier-Daire V. (2009). The mutation spectrum in RECQL4 diseases. Eur J Hum Genet.

